# Recent Developments in Enzyme, DNA and Immuno-Based Biosensors

**DOI:** 10.3390/s18061924

**Published:** 2018-06-13

**Authors:** Melis Asal, Özlem Özen, Mert Şahinler, İlker Polatoğlu

**Affiliations:** 1 Department of Molecular Biology and Genetics, İzmir International Biomedicine and Genome Institute, Dokuz Eylül University, Balçova, 35340 İzmir, Turkey; melis.asal@ogr.deu.edu.tr; 2 Department of Pharmacology, Ege University, Institute of Health Sciences, Bornova, 35040 İzmir, Turkey; zlemozen@gmail.com; 3 Department of Bioengineering, Graduate School of Natural and Applied Sciences, Ege University, Bornova, 35040 İzmir, Turkey; mertsahinler@hotmail.com; 4 Bioengineering Department, Manisa Celal Bayar University, Yunusemre, 45140 Manisa, Turkey

**Keywords:** biosensor, immunosensor, enzyme, DNA, medical analysis, environmental analysis, food analysis, nanotechnology

## Abstract

Novel sensitive, rapid and economical biosensors are being developed in a wide range of medical environmental and food applications. In this paper, we review some of the main advances in the field over the past few years by discussing recent studies from literature. A biosensor, which is defined as an analytical device consisting of a biomolecule, a transducer and an output system, can be categorized according to the type of the incorporated biomolecule. The biomolecules can be enzymes, antibodies, ssDNA, organelles, cells etc. The main biosensor categories classified according to the biomolecules are enzymatic biosensors, immunosensors and DNA-based biosensors. These sensors can measure analytes produced or reduced during reactions at lower costs compared to the conventional detection techniques. Numerous types of biosensor studies conducted over the last decade have been explored here to reveal their key applications in medical, environmental and food industries which provide comprehensive perspective to the readers. Overviews of the working principles and applications of the reviewed sensors are also summarized.

## 1. Introduction

A biosensor is a device that can detect physiologic or biochemical changes by incorporating biological and physiochemical components. It mainly consists of a biomolecule, a transducer and an output system. The specificity of the biosensors mainly relies on the used biomolecule, which can be enzymes, nucleic acids, antibodies, cells and tissues. The extreme selectivity of biomolecules leads to recognition of analytes and the biochemical signal produced during recognition is converted to a detectible signal by the transducer. The signal is usually displayed as an electrical signal by the output system.

Biosensors are designed in a variety of sizes and shapes and can employ a range of transducers such as electrochemical, optical, piezoelectric, thermal or ion-selective electrodes. Among these transducers electrochemical and optical ones are mostly preferred due to their characteristics advantages [1]. Electrochemical transducers can sense produced or consumed ions or electrons as a result of a reaction between the target analyte and biomolecule. Amperometric, potentiometric and impedimetric methods can be employed in electrochemical biosensors [2,3]. In amperometric biosensors, the potential between the two electrodes is set and the current produced by the oxidation or reduction of electroactive species is measured and correlated to the concentration of the analyte [4]. In potentiometric biosensors, the signal is generated through the difference in ion concentration between ion selective electrodes. The signal can be correlated to the amount of target analyte concentration [5]. In electrochemical impedance spectroscopy (EIS), the frequency-domain response can give useful data about the physical and chemical changes that occur when an analyte binds to a biomolecule immobilized on an electrode. Moreover, it does not require any label and can monitor the binding affinity in real time [3].

Optical biosensors respond to a target analyte in the form of an identifiable output signal generated through changes in optical characteristics of the analyte. These biosensors can be utilized in medical diagnostics, environmental and food screening due to showing relatively high immunity to electromagnetic interference, simplicity of the measurement procedure and small-sized instrumentation [6]. Surface Plasmon Resonance (SPR) is a sensitive and label free method that is used to detect biomolecular interactions occurring in close proximity to the transducer surface. SPR components include the light source, gold film, a prism, the biomolecule, flow system and a detector. The resonance conditions are highly affected by the biomolecules that are immobilized on the gold transduction surface. Any conformational change in biomolecules leads to a change in the refractive index and is monitored as a shift in the resonance angle, which gives information about the amount of the target substrate [7]. Evanescent wave based sensing systems are favored over other optical sensors in some situations because of the restriction of the interaction between the light and fluidics pathway to single interface, which provides a higher flexibility in the general sensor design [8].

Nanotechnology provides a promising way to design electrochemical and optical biosensors. The use of nanocomposites or inorganic nanoparticles such as nanotubes, nanowires and nanorods in electrodes for their great conductivity and catalytic properties is a wise way to improve sensor performance [9]. These nanoparticles and their composite structure are widely employed as recognition biomolecules in order to improve speed, sensitivity and biocompatibility of these highly selective biosensor devices.

Overall, biosensors are likely to be preferred over conventional methods due to their ease of use and compactness. Since the discovery of the oxygen electrode by Clark, many biosensors have become commercial products, owing to their ability to provide rapid detection of analytes in diverse fields such as medical diagnostics, environmental monitoring, food processing and industrial processes [10]. Numerous significant studies covering clinical diagnostics and screening, environmental and food screening and monitoring applications about enzymatic biosensors, DNA-based biosensors and immunosensors conducted over the last decade were arranged in this paper to represent an overview of the novelties in the area to the readers.

## 2. Enzymatic Biosensors

**Figure 1:**
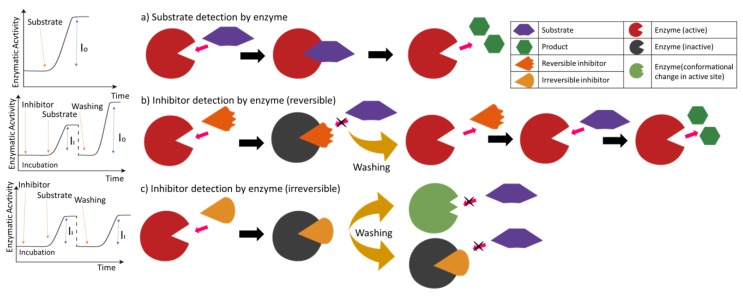
Scheme of enzymatic biosensor for substrate and inhibitor (reversible and irreversible) detection.

Biosensors that work based on the relationship between an enzyme and its substrate are referred to as enzymatic biosensors. This type of biosensors work on two main mechanisms depending on target analyte; substrate detection and enzyme inhibition as illustrated in Figure 1. Substrate detection mechanisms are based on the conversion of the substrate by an enzyme incorporated in the biosensor. On the other hand, the working principle of inhibition based enzymatic biosensors (IBEBs) lies in the ability of the target analyte to reduce enzymatic activity. Enzyme inhibition method is based on the determination of enzyme activity in the presence and absence of inhibitor compounds [11]. The decrease in product concentration provides the detection of inhibitory targets that inhibit the activity of certain enzymes.

Enzymatic biosensors can incorporate electrochemical, optical, and calorimetric transducers. Among these transducers, electrochemical ones are the type most commonly used in literature, probably due to the first developed biosensor being an enzymatic electrochemical biosensor (Clark) [12]. This type of biosensors is widely used in medical, environmental, food, agricultural and pharmaceutical industries. In addition to being cost effective, they are known to offer fast results [9].

As a medical application, Hernández-Ibáñez et al., 2016 [13] developed a sensitive, easily manufactured and stable novel amperometric biosensor to monitor lactate levels in embryonic cell cultures. In this study, disposable screen-printed electrodes are utilized as the sensing platform. Chitosan/multi-walled carbon nanotubes composite are used for the immobilization of lactate oxidase onto the electrode surface. The production of pyruvate and H_2_O_2_ as a result of the reaction of lactate oxidase with lactate leads to a series of reactions. The final complex’s reduced oxidized state is measured by CV and CA. The biosensor is shown to provide excellent sensitivity along with simple and fast detection [13]. Another biosensor was developed by Mansor and friends [14] in 2018 for use in human samples to rapidly diagnose bacterial sepsis. This tri-enzyme amperometric biosensor offers easy detection of secretory phospholipase Group 2-IIA (sPLA2-IIA), a sepsis and bacterial infection biomarker. The immobilization of the choline kinase, choline oxidase and horseradish peroxidase enzymes on acrylic microspheres is accomplished by utilizing succinimide groups. In this sensor, the reaction between sPLA2-IIA and its substrate leads to a cascade reaction and then to a detectible H_2_O_2_ generation. Human sample analyses showed the usability of this biosensor for point-of-care detections with good reproducibility [14]. In another approach, a printed bi-enzyme electrochemical sensor was established by Ahmadraji et al., 2017 [15] to measure total cholesterol in serum. The measurement is conducted by following the reduction of hydrogen peroxidase by the cholesterol esterase and cholesterol oxidase enzymes immobilized on a silver paste electrode. In order to increase the electro-catalytic activity of the sensor, Triton X-100 is used. The sensor is able to detect the total cholesterol in a serum in the diagnostic range through an amperometric measurement [15]. Alvi et al., 2013 [16] developed a potentiometric biosensor using lnN/lnGaN QDs to measure cholesterol concentration for diagnostic applications. As opposed to Ahmadraji’s study, in this sensor only cholesterol oxidase is used for detection. The developed sensor shows high sensitivity with a rapid output response time and logarithmic concentration range. Also, the sensor with lnN QD and a sensor with lnN thin film are compared in terms of response time and sensitivity. It is shown that the lnN QD based biosensor is twice more sensitive and five times faster than lnN thin layer based sensor [16].

Enzymatic biosensors have also found environmental applications. Fang and colleagues [17] developed an amperometric sensor to minimize crop damages by detecting methyl salicylate, which is released by plants in the case of infections. The sensor is based on a bi-enzyme modified electrode. The detection occurs through a cascade of reactions resulting in an amperometric signal. CV and CA tests establish higher sensitivity and a lower detection limit than the preexisting sensors. The sensor also shows great durability and repeatability without being affected by the presence of other compounds [17]. A novel enzymatic, carbon nanotube and gold nanowires-based biosensing platform was developed by Kurbanoğlu et al., 2018 [18] to detect catechol. This phenolic compound is released into the environment during its manufacturing and use. Because of catechol’s oxidizable properties, the cascade of reactions that the catechol leads in the presence of tyrosinase can be amperometrically monitored. Tyrosinase is an oxidoreductase which catalyzes the transfer of electrons. Hence, the detection principle of the sensing platform lies on the rapid electron transfer between immobilized tyrosinase and the transducer. Sensitive detection of catechol is achieved due to the synergetic effect of carbon nanotubes and gold nanowires [18]. In another study, a gold nanoparticle-based electrochemical biosensor employing tyrosinase was developed to detect catechol by Kızılkaya et al., 2017 [19]. The sensor was shown to be highly sensitive with a wider linear range and a lower LOD compared to the sensor developed by Kurbanoğlu et al. This sensor is a promising candidate for cheap, fast, and easy detection of phenolic compounds [19].

The use of enzymatic biosensors is of great importance in the food industry. Ibupoto and colleagues [20] established an l-ascorbic acid biosensor in 2011, due to l-ascorbic acid levels of foods being important for the human health. In this sensor, ascorbate oxidase enzyme is immobilized on ZnO nanorods by cross linking with 3-glycidoxypropy1trimethoxysilane (GPTS). A wide linear range and good sensitivity is observed, and the biosensor shows fast response time, good selectivity, reproducibility and no significant interference of common ions [20]. In another study, an electrochemical biosensor to detect tyramine in food samples was developed by Sánchez-Paniagua López et al., 2017 [21]. Detection of tyramine is highly important since it can be used as a quality indicator for foods. The design of the biosensor involves tyrosinase immobilization into orthophosphate calcium matrices by using glutaraldehyde. The sensor utilizes the amperometric measurement techniques to detect tyramine as a result of the electrochemical reduction of the o-dopaquinone. The developed biosensor provides a highly sensitive, rapid and inexpensive way to detect the tyramine along a wide linear range, with high sensitivity, and with a low detection limit [21]. In 2015, Ang et al. [22] developed an electrochemical biosensor to measure glucose content of fruits. The sensor uses glucose oxidase enzyme immobilized on chitosan via cross linking with glutaraldehyde. Under applied potential, the electrode’s amperometric response is proportional to H_2_O_2_ concentration. Since H_2_O_2_ is a side product of oxidation reaction of glucose, it is possible to determine glucose concentration through its generation with this sensor [22]. A somewhat simple class of sensing techniques has been developed in the paper and plastic-based forms compatible with enzymatic biosensors [9]. These inexpensive assays have been combined with both colorimetric and electrochemical detection methods [23,24]. A novel paper-based amperometric enzymatic biosensor platform for glucose detection in food samples was developed by Amor-Gutiérrez et al., 2017 [25]. The platform was used as a clip to support the paper which was sandwiched between the working electrode and the reference and auxiliary electrodes. The fabrication of this biosensor is cheap and quick. Moreover, the sensor provides a wide linear range, small sample volume requirement and easy measurement [25]. 

Another example which displays the simplicity, cost-effectiveness, and rapidness of paper-based biosensors was developed by Suaifan et al., 2017 [26] that detects *Staphylococcus aureus*. The sensing occurs through the proteolytic activity of *S. aureus* proteases on a specific peptide substrate. This biosensor was proposed for utilization on various samples such as clinical isolates, environmental samples and food matrices. Moreover, cost-effectiveness and convenience combined with the high sensitivity make this biosensor a powerful sensing platform for the detection of *S. aureus* [26].

The analytical performances of these enzymatic biosensors are summarized in Table 1.

Numerous methods based on enzyme inhibition are also available in the literature. Among other enzymes, acetylcholinesterase (AChE) is the most commonly used biomolecule in inhibition based enzymatic biosensors (IBEBs) due to its high turnover number, affordable cost and its widely distributed inhibitors in the environment [27]. IBEBs have been reported to be useful for detection of pesticides, organophosphorus mycotoxins and some other compounds [28]. The degree of inhibition can be quantified using the following formula where I_0_ and I_1_ denote the enzyme activity in the absence and presence of inhibitor compounds [12].

$$\mathrm{Degree}\;\mathrm{of}\;\mathrm{Inhibition}\;=\frac{\left(I_{0}-I_{1}\right)}{I_{0}}\times 100$$

A selection of recent publications about the AChE IBEBs used for detection of pesticide as environmental pollution and food contaminants are given in Table 2. Still, despite the vast amount of studies present in the literature, most of them suffer from the poor selectivity due to the fact that inhibition can occur due to these compounds [28].

## 3. DNA-Based Biosensor

**Figure 2:**
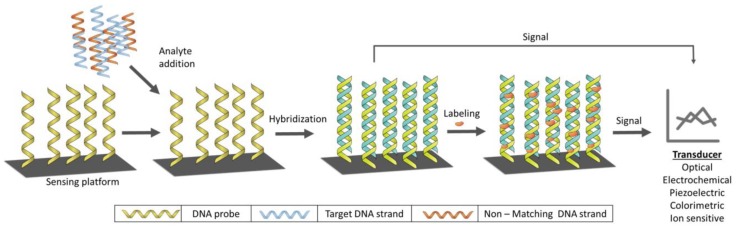
General design and working principle of DNA biosensor.

The principle of these widely used sensors lies in the hybridization process through spontaneous hydrogen bonding between the target DNA and its complementary strand. This principle is usually utilized by immobilizing the single-stranded DNA (ssDNA) onto a suitable surface [38]. The hybridization event is generally detected by two different methods; (i) the detection of certain electroactive indicator (labeling) (ii) the detection of signal produced by the most electroactive base of DNA [39] as depicted in Figure 2.

DNA-based biosensors are employed in the clinics to detect specific mutations. A novel biosensor using hematoxylin as an electrochemical label was developed by Aghaei et al., 2017 [40]. Screen-printed gold electrode (SPGE) with thiolated DNA probes was used to detect the point mutation of the phenylalanine hydroxylase (PAH) enzyme, which is the cause of Phenylketonuria. The performance of the sensor was investigated by using CV and EIS, and the selectivity was tested using 1 and 3 mismatched oligonucleotides in comparison to the complementary DNA. Despite being structurally simple, the device displayed great sensitivity and selectivity in real samples [40]. DNA-based biosensors are also designed for vital purposes such as detection of pathogens. An electrochemical DNA biosensor based on Ni doped ZnO thin film was developed by Tak and his friends (2017) [41] in order to detect the bacterial agent of the fatal disease meningitis. In this study, DNA probes were immobilized on the Ni-ZnO/ITO electrode surface under favor of the electrostatic interaction between probes and the surface. The results were obtained by using and the developed sensor appears to be cheaper, faster, less invasive and more selective than the currently used methods [41]. Another electrochemical DNA biosensor was developed by Shakoori et al., 2014 [42] to detect hepatitis B virus (HBV). Gold nanorods were utilized in the sensor surface to increase the total surface area for better immobilization of the ssDNA. The sensor shows promise for the detection of HBV in serum samples [42]. In a 2018 paper, another biosensor for label free HBV detection was introduced by Shariati [43]. This work is about an ITO nanowires based DNA biosensor compatible with metal-oxide-semiconductor (CMOS) technology. The sensor integrates field effect transistor (FET). The immobilization of thiolated HBV ssDNA is supported with the employment of ITO nanowires. The analyses showed that the DNA-based biosensor is able to discriminate between the non-complementary, mismatch and complementary sequences. This sensor is highly promising in HBV detection due to possessing a much lower LOD and a significantly wider linear range compared to the study conducted by Shakoori et al., 2014 [42,43]. The first label-free user-friendly electrochemical DNA biosensor for detection of hepatitis A virus (HAV) was developed by Manzano et al., 2018 [44]. The detection occurs through the hybridization reaction that leads to an electrochemically detectable change in the signal. The hybridization reaction occurs on the disposable gold electrode functionalized with specific capture probes. The measurements are monitored through the oxidative peak potential by using cyclic voltammetry. The rapidness, cost-effectiveness and use of ease of the developed biosensor are reported [44]. Another pathogen that can be detected via biosensors is the *Brucellosis* agent. Sattarahmady and his friends (2015) [45] designed an optical biosensor to detect the presence of genomic DNA of *Brucella* spp. in clinical samples. The working principle of this sensor relies on a specific oligonucleotide electrode combined with Au nanoparticles. The presence of the complementary strand results in the hybridization, and is detected by spectroscopic measurements. Additionally, the sensor is less time consuming and is unique in this field in terms of its low cost and rapid detection [45]. Cellulose fiber paper devices to be used for medical tests have been developed with the purpose of producing practical DNA-based biosensors. Lu and his friends (2012) [46] produced a DNA electrochemical biosensing platform based on a 3D folding paper device for point of care testing using screen-printed electrodes modified with graphene to provide faster electron transport, high thermal conductivity, and biocompatibility. Au nanoparticles were combined with the electrodes to create a synergic interaction with graphene. This combination provided a faster electronic transmission rate and ahigher surface area, while creating an immobilization matrix for ssDNAs to keep them highly stable and active. Thionine was used as a signaling molecule for dsDNAs and showed high sensitivity. According to results of this study, the developed device is proved to be sensitive and has potential use in human serum sample tests [46].

In addition to pathogen detection, DNA-based optical biosensors can be effectively implemented in real-time detection of trace amounts of heavy metals in the environment [47]. A rapid optical DNA biosensor for in-situ detection of heavy metal ions was developed by Long et al., 2013 [47]. In this sensor, the immobilized DNA probes bind to the introduced fluorescence-labeled Thymine (T)-rich DNA structure. The working principle of the system utilizes the ability of the heavy metals to interact with the thymine bases and to form the T-Heavy metal-T complex that creates a hairpin structure which leads to the dehybridization of the structure from the sensor surface. Detection of Hg^2^+ by using this structure-switching DNA sensing technique proved to have a decent sensitivity, portability and speed [47]. A novel electrochemical DNA biosensor including Au nanoparticles was developed by Zhang and his friends (2017) [48] in order to sensitively detect Hg^2+^, a potent toxin. Studies proved that the thymine base of DNA could selectively and sensitively bind to Hg^2+^. In addition to the sensor’s good selectivity and stability, it was shown to be able to work in water samples [48]. An impedimetric DNA biosensor to detect Ag^1+^ ions was developed by Liu et al., 2014 [49]. The system utilizes the ability of Ag^1+^ to interact and stabilize the cytosine-cytosine mismatch. An amplified impedance signal was obtained due to the usage of Hemin/G-Quadruplex nanowire in the system. The combination of the impedimetric technique with the Hemin/G-Quadruplex nanowire was proved to be useful in achieving high sensitivity and selectivity [49].

More recently, a DNA biosensor aimed to detect pathogens in food samples was developed by Xu and colleagues (2017) [50] against *Escherichia coli* O157:H7 to overcome outbreaks of infection. The sensor was based on graphene oxide/chitosan nanocomposites modified glassy carbon electrode and showed a wide detection range and low detection limit for target ssDNA based on EIS studies. Furthermore, it was able to distinguish between complementary DNA and 1 and 2 base mismatched DNA molecules by the DPV technique [50]. A fiber-optic DNA based biosensor was developed for detection of Listeria monocytogenes, a bacterium that can cause blood stream infections, gastroenteritis or even abortion, from food products by Ohk et al., 2010 [51,52]. The sensor utilizes a reporter molecule, single-stranded oligonucleotide ligand specific for L. monocytogenes, and the capture molecule anti-Listeria antibody. The combination of antibody and oligonucleotide in the sensing system increases the possibility of selective detection. The developed sensor successfully detects *L. monocytogenes* from artificially contaminated food samples [51]. In another study, Surface-enhanced Raman scattering (SERS) spectroscopy based nanosensor was developed to detect genetically modified rice expressing insecticidal proteins by Chen et al., 2012 [53]. The sensor utilizes SERS-barcoded nanoparticles, which are target specific oligonucleotide strands conjugated to gold nanoparticles encapsulated with silica. The comparable results by means of sensitivity and accuracy of the developed sensor with the real-time PCR were reported by the authors.

Table 3 presents a selection of the most recent publications on DNA-based biosensors.

## 4. Immunosensors

**Figure 3:**
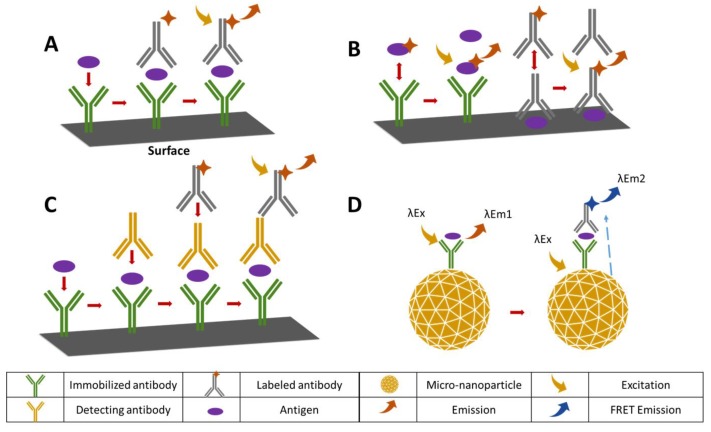
Possible immunoassay binding configurations: (**A**) sandwich structure formation (**B**) competitive style immunoassays (**C**) extended sandwich structure formation (**D**) sandwich structure formation on a (micro-nanoparticle) surface.

Immunosensors have emerged as a powerful tool in clinical diagnostics, environmental monitoring and food safety applications due to their extreme specificity [54]. The transducers of these sensors contain antibodies immobilized through covalent interactions by introducing functional groups such as carboxyl, amino, aldehyde, or sulfhydryl [55,56]. The working principle is based on detecting, processing and displaying the signal caused by the formation of an antibody-antigen (Ab-Ag) complex [54]. Figure 3 depicts some possible immunoassay binding configurations.

Immunosensors are mainly used in clinics for disease detection and monitoring. The first amperometric immunosensor to detect TGF-β1 in urine samples was developed by Sánchez-Tirado et al., 2017 [57]. This immunosensor provides the sensitive, reliable and robust detection of TGF-β1, a biomarker related to renal disease, by utilizing functionalized magnetic microparticles immobilized onto a screen-printed carbon electrode. This immunosensor utilizing the sandwich-type immunoassay technique has proved its usefulness by detecting low concentrations of TGF-β1 in real samples [57]. An electrochemical immunosensor based on functionalized nitrogen doped graphene QD was developed by Yang and friends (2017) [58] in order to detect the cancer biomarker carcino-embryonic antigens (CEA) in human serum. Nitrogen doped graphene quantum (N-GQD), PtPb nanoparticles and Au nanoparticles were combined to get better electrochemical activity from the reduction reaction and to provide more surface area. As a result of this study; a sensitive, selective and stable assay was developed [58]. Optical transducers are widely implemented in clinical and environmental immunochemistry due to their advantages, including fast signal generation and rapid reading times [59]. An immunosensor based on photonic crystal fibers for detection of alpha fetoprotein (AFP) was developed by Liu and friends (2017) [60]. AFP is one of the most significant tumor markers of hepatocellular carcinoma. In the presence of the AFP antigen–human antibody interaction, laser induced fluorescence detection can be achieved. This immunosensor provides a large surface area to volume ratio. The sensor sensitivity is 35 times better than ELISA in regard to LOD. Additionally, detection is reproducible and the signal to noise ratio is lower than other methods [60]. An electrochemical immunosensor using nickel oxide (NiO) thin film was developed by Kaur et al., 2017 [61] for the label free detection of total cholesterol and low density lipoprotein (LDL) levels in serum samples. The thin film was used for the covalent immobilization of apolipoprotein B-100 antibody. The sensor was assayed using differential pulsed voltammetry (DPV), CV and EIS. The impedimetric studies showed that the immunosensor was able to detect the Ab-Ag complex formation sensitively over a wide linear range. The sensor holds great promise for detection in the blood serum, with additional long shelf life and regeneration ability advantages [61]. In another study, a voltammetric immunosensor for detection of survival motor neuron (SMN) 1 gene was developed with the purpose of spinal muscular atrophy screening. The immunosensor utilizes a carbon nanofiber-modified screen-printed electrode covalently functionalized with 4-carboxyphenyl. The SMN antibodies are immobilized via carbodiimide hydrochloride (EDC)/*N*-hydroxysuccinimide (NHS) to the electrode surface. Sensitiveness of SMN detection, cost-effectiveness and simplicity of the developed sensor is reported [62]. In 2018, Wang and friends [63] introduced a label-free, paper-based electrochemical immunosensor combining microfluidics and nanotechnology for point-of-care detection of 17β-estradiol, a reproduction regulator in humans. The screen-printed working electrode is coated with the nanocomposite consisting of multi-walled carbon nanotubes, thionine and gold nanoparticles for immobilization of the antibody anti-E2 and to serve as electrochemical mediators and electron transfer accelerators for signal amplification. The sensor is shown to be capable of sensitive and lost cost detection of 17β-estradiol at the point-of-care [63].

Environmental applications of immunosensors include detection of unwanted compounds in water samples. McNamee et al., 2013 [64] developed a SPR based immunobiosensor to detect harmful phycotoxin domoic acid (DA). The developed sensor requires a sample preparation and detects the domoic acid in 13 min. The sensor is proposed to be utilized as an early warning monitoring system by the authors [64]. More recently, Another SPR based immunobiosensor to detect DA was developed by Colas et al., 2016 [65]. This sensor utilizes a specific system consisting of a light source, a spectrophotometer, optics and two SPR chips. The sensor successfully detects domoic acid upon deployment in the seawater with a much lower LOD compared to the sensor developed by McNamee. The sensor is therefore promising to be used as a novel tool for in-situ analysis in seawater [65]. Xiao-hong et al., 2014 [8] developed an optical immunosensor using evanescent wave for detection of Bisphenol A (BPA) in water samples. An easy and rapid detection method for BPA is required due to BPA being a harmful chemical that can mimic hormones. In this novel biosensor, fluorophore-tagged antibodies are excited due to evanescent wave field formation. The constructed biosensor showed high sensitivity, selectivity and reusability [8].

Immunosensors play an important role in detection of hazardous substances in foods. A simple and rapid, gold nanorods based label-free optical biosensor for the detection of aflatoxin B1, which is a highly dangerous chemical found in foods, was developed by Xu et al., 2013 [66]. Gold nanorods were utilized as the sensing mechanism due to their ability to provide high stability under high ionic strength and detection of the interested molecule was provided through antibodies. False results occurring from undesirable aggregations were effectively reduced by utilizing the competitive dispersion principle. The optical nano-biosensor was confirmed to be highly selective, sensitive and simple [66]. Wang and his friends (2017) [67] developed a label free SPR based immunosensor to detect ractopamine in swine urine. The sensor was developed to overcome the risk of ractopamine consumption since it is harmful to the human cardiovascular and central nervous systems. The design of this immunosensor includes a secondary antibody as an addition to the primary antibody with the purpose of increasing the performance of the sensor. The developed sensor proved to have high sensitivity, good stability and selectivity. Also, the comparison of the SPR immunosensor with a traditional indirect competitive ELISA in terms of LOD, analysis time and reagent consumption demonstrated the superiority of the SPR immunosensor [67]. An impedimetric label-free immunosensor to detect bacterial pathogens from whole milk was developed by Joung et al., 2013 [68]. The sensor utilizes a commercial alumina nanoporous membrane functionalized with hyaluronic acid (HA) along with an electrochemical transducer. The developed immunosensor successfully detected *E. coli* O157:H7 from whole milk. Therefore, the sensor can be used as an early pathogen detection tool from milk sample [68].

An electrochemical immunosensing platform based on concave gold nanocuboids (CAuNCs) was developed for the label free detection of Ag-Ab interactions. The CAuNCS caused a redox current response in the CV studies. Upon the introduction of model IgG antigens, the anodic peak current reduced remarkably due to the formation of the Ag-Ab complex [69]. Another immunosensing platform was developed by Bi & Yang, 2010 [70] using the optical properties of liquid crystals. Detection of anti-FLAG M2, a model antibody, was performed by building a sensitive layer at the liquid crystal-aqueous interface using immobilized linear oligopeptides. The change in the optical texture of liquid crystals as a result of the binding allowed the detection of anti-FLAG M2 [70].

Some of the recent publications about the immunosensors are given in Table 4.

## 5. Conclusions

The use of biosensors in easy, fast and low cost detection of complex substances has increased their value economically and socially. In the past 56 years, numerous studies have been published regarding development of the enzymatic biosensor, the DNA-based biosensor and immunosensors for use in a range of fields. However, only a small percentage of these studies were realized commercially.

Nanotechnology, which is one of the key technologies of the 21st century, has made a significant contribution to biotechnology with the fabrication of novel nanomaterials with important applications in biosensors. In particular, magnetic nanoparticles, carbon nanotubes, and quantum dots have found their places in the biosensor field. Gold nanoparticles in different shapes are utilized frequently in electrochemical biosensors and optical biosensors employing SPR. Utilization of nanomaterials enhance magnetic, optical, as well as electrochemical properties of biosensors. Although the fabrication and characterization of desired nanomaterials and understanding the behaviors of these materials on electrode surfaces are still challenging, nanobiosensors show great promise for point of care detection applications by utilizing bioengineering approaches.

With the help of advances in nanotechnology, biochip technology and microfluidics, novel commercial biosensors for applications in medical, environmental and food industries will soon take their places at the markets. Taking the growing interest into account, in the near future the biosensor technology will show superiority in sensitivity, measurement time, cost and automation over conventional physicochemical testing devices. Thus, the development of reliable and low-cost biosensors has become more crucial now than ever, and development of the perfect biosensors does not seem to be far away.

## Figures and Tables

**Table 1:** Analytical performances of recent publications on enzymatic biosensors.

Sensor	Target	LOD	Linear Range	Sensitivity	References
**Medical Applications**					
Chitosan/Multi-Walled Carbon Nanotubes Based Amperometric Biosensor	Lactate	0.0226 mM	0.0304–0.243 mM	3417 ± 131 μA·M^−1^	[13]
Tri-Enzyme Amperometric Biosensor	sPLA2-IIA	5 × 10^−3^ ng/mL	0.01–100 ng/mL	N/A	[14]
Printed H_2_O_2_ Based Amperometric Sensor	Total cholesterol	2 mM	60 U/mL cholesterol oxidase	0.0224 µA/mM	[15]
QD Based Potentiometric Biosensor	Cholesterol	N/A	0.001–1 mM	97 mV/decade	[16]
**Environmental Applications**					
Bi-Enzyme Modified Electrode Based Amperometric Sensor	Methyl salicylate	CV: 0.02295 mM CA: 0.00098 mM	CV:0–1.0 mM CA:0–0.1 mM	CV: 112.37 CA: 282.82 μA·cm^−2^·mM^−1^	[17]
Carbon Nanotube, Gold Nanowires And Tyrosinase Based Amperometric Biosensor	Catechol	27 × 10^−6^ mM	0.0005–0.042 mM	N/A	[18]
Gold nanoparticle and Tyrosinase based Amperometric Biosensor	Catechol	13.8 × 10^−6^ mM	0.046 × 10^−3^–0.05 mM	1.144 µA/µM	[19]
**Food** **Applications**					
Zno Nanorods Based Potentiometric Bioensor	l-Ascorbic Acid	0.001 mM	0.001–50 mM	32 mV/decade	[20]
Amperometric Biosensor	Tyramine	4.85 × 10^−5^ mM	0.00058–0.016 mM	1.50 × 10^3^ mA·M^−1^·cm^−2^	[21]
Amperometric Biosensor	Glucose	0.05 mM	N/A	N/A	[22]
Paper-Based Enzymatic Bioensor	Glucose	0.12 mM	0.3–15 mM	1.13 µA/mM	[25]
**General Applications**					
Paper-Based Optical Biosensor	*S. aureus*	7 CFU/mL in pure broth culture, 40 CFU/mL in food matrices, 100 CFU/mL in environmental samples	N/A	N/A	[26]

**Table 2:** Detection of pesticide by acetylcholinesterase inhibition based enzymatic biosensors (CA: Chronoamperometry, CV: Cyclic voltammetry, DPV: Differential Pulse Voltammetry).

Analyte	Detection Method	LOD/LOQ	Storage Stability (4 °C)	Reference
Malathion	CV	0.3 nmol·L^−1^	N/A	[29]
Dichlorvos	DPV	29 nM (6.4 ppb)	N/A	[30]
Paraoxon	CV	3.6 pM	After 30 days at 80%	[31]
Malathion	CA	1 fM	After 30 days at 89%	[32]
Methamidophos, Chlorpyrifos	DPV	1 µg·L^−1^ 0.05 µg·L^−1^	After 30 days at 80%	[33]
Paraoxon, Dimethoate	CV	0.7 nM 3.9 nM	After 30 days at 93%	[34]
Carbaryl	CA	20 ng·mL^−1^	After 15 days at 80.6%	[35]
Malathion, Carbaryl	CA	4.14 pg·mL^−1^ 1.15 pg·mL^−1^	After 28 days at 92%	[36]
Carbaryl Monocrotophos	DPV	5.3 fM 0.46 fM	After 15 days at 95.2%	[37]

**Table 3:** Analytical performances of recent publications on DNA-based biosensors.

Sensor	Target	LOD	Linear Range	Sensitivity	Reference
**Medical Applications**					
Electrochemical Biosensor Based on Screen-Printed Gold Electrode And Hematoxylin	Point mutation IVS10nt-11g→a of the PAH gene	0.0085 nM	0.02–150 nM	N/A	[40]
Electrochemical Biosensor Based on Ni Doped Zno Thin Film	A specific sequence of *Neisseria meningitidis* DNA	5 ng/μL (707 nM)	5–200 ng/μL (707–28 × 10^3^ nM)	49.95 μA/decade	[41]
Amperometric Biosensor Based on Gold Nanorods	HBV virus ssDNA	0.002 nM	0.001–1 × 10^4^ nM	N/A	[42]
FET Based Biosensor Modified with ITO Nanowires	HBV ssDNA	1 fM	1 fM to 10 μM	N/A	[43]
Amperometric Biosensor	A specific sequence of Hepatitis A virus	0.00065 nM	0.01–10 pg/μL	N/A	[44]
Optical Biosensor	*Brucella* spp. genomic DNA	1.09 ng/mL (0.1457 nM)	1.36–102.5 ng/mL (0.2–13.7 nM)	3.94 OD µL·ng^−1^	[45]
Amperometric Biosensor Based on A 3D Folding Paper Device	Target ssDNA	2 × 10^−16^ mmol·L^−1^ (2 × 10^−10^ nM)	8 × 10^−7^–0.5 nM	N/A	[46]
**Environmental Applications**					
Structure-Switching Based Optical Biosensor	Hg^2+^	1.2 nM	N/A	N/A	[47]
Au Nanoparticles Functionalized Electrochemical Sensor	Hg^2+^	0.05 nM	0–200 nM	N/A	[48]
Impedimetric Biosensor for Ag^+^ Detection	Au	0.05 nM	0.1–1 × 10^5^ nM	N/A	[49]
**Food Applications**					
Impedimetric DNA Biosensor Based On Graphene Oxide/Chitosan Nanocomposites	Complementary DNA sequence specific to *E. coli* O157:H7	3.5 × 10^9^–15 × 10^9^ nM	1 × 10^−5^ nM	N/A	[50]
Fiber-Optic DNA Biosensor Based On Aptamer And Antibody	Aptamer specific for internalin A of *L. monocytogenes*	1 × 10^3^ CFU/mL	N/A	N/A	[51]
SERS based genetically modified rice biosensor	*Bacillus thuringiensis* oligonucleotide strands	0.0001 ng/mL	0.001–10 ng/mL	N/A	[53]

**Table 4:** Analytical performances of recent publications on immunosensors.

Sensor	Target	LOD	Linear Range	Sensitivity	Reference
**Medical Applications**					
Amperometric Immunosensor	TGF-β1	0.01 ng/mL in urine	0.015–3 ng/mL	0.06 ng/mL	[57]
Functionalized N-GQD Based Amperometric Immunosensor	CEA	0.000002 ng/mL	5 × 10^−6^–50 ng/mL	N/A	[58]
Photonic Crystal Fiber-Based Optical Immunosensor	AFP antigen	0.1 ng/mL	0.1–150 ng/mL	N/A	[60]
Amperometric Immunosensor Using Nio Thin Film	LDL	15 nM	18–500 nM	12 kΩ·μM^−1^	[61]
Carbon Nanomaterial-Modified Electrode Based Amperometric Immunosensor	SMN protein	0.00075 ng/mL	0.001–100 ng/mL	N/A	[62]
Paper-Based Amperometric Microfluidic Immunosensor Modified with Nanocomposites	17β-estradiol	10 pg·mL^−1^	0.01–100 ng·mL^−1^	N/A	[63]
**Environmental Applications**					
SPR based optical immunosensor	DA	1.66 ng/mL	N/A	N/A	[64]
SPR based optical in-situ immunosensor	DA	0.1 ng/mL	0.1–0.2 ng/mL	N/A	[65]
Optical Evanescent Wave Immunosensor	BPA	30 ng/mL	124–9600 ng/mL	N/A	[8]
**Food Applications**					
Gold Nanorod Based Optical Biosensor	Aflatoxin B1	0.16 ng/mL	0.5–20 ng/mL	N/A	[66]
Label-Free Optical Immunosensor	Ractopamine	0.009 ng/mL	0.3–32 ng/mL	N/A	[67]
Impedimetric label-free *E. coli* immunosensor	Anti-*E coli* O157:H7 antibodies	8.3 × 10^1^ CFU/mL	0–105 CFU/mL	N/A	[68]
**General Applications**					
Concave Gold Nanoparticle-Based Amperometric Immunosensor	Ag-Ab interactions	5 ng/mL	10–200 ng/mL	N/A	[69]
Liquid Crystal Based Optical Immunosensor	Anti-FLAG M2	27 ng/mL	N/A	60 ng/mL	[70]
